# Mechanical force antagonizes the inhibitory effects of RecX on RecA filament formation in *Mycobacterium tuberculosis*

**DOI:** 10.1093/nar/gku899

**Published:** 2014-10-07

**Authors:** Shimin Le, Hu Chen, Xinghua Zhang, Jin Chen, K. Neelakanteshwar Patil, Kalappa Muniyappa, Jie Yan

**Affiliations:** 1 Mechanobiology Institute, National University of Singapore, Singapore 117411, Singapore; 2 Department of Physics, National University of Singapore, Singapore 117542, Singapore; 3 Department of Physics, Xiamen University, Xiamen 361005, China; 4 BioSystems and Micromechanics (BioSyM) IRG, Singapore-MIT Alliance for Research and Technology, National University of Singapore, Singapore 138602, Singapore; 5 Department of Biochemistry, Indian Institute of Science, Bangalore 560012, India; 6 Centre for Bioimaging Sciences, National University of Singapore, Singapore 117557, Singapore

## Abstract

Efficient bacterial recombinational DNA repair involves rapid cycles of RecA filament assembly and disassembly. The RecX protein plays a crucial inhibitory role in RecA filament formation and stability. As the broken ends of DNA are tethered during homologous search, RecA filaments assembled at the ends are likely subject to force. In this work, we investigated the interplay between RecX and force on RecA filament formation and stability. Using magnetic tweezers, at single molecular level, we found that *Mycobacterium tuberculosis* (Mt) RecX could catalyze stepwise de-polymerization of preformed MtRecA filament in the presence of ATP hydrolysis at low forces (<7 pN). However, applying larger forces antagonized the inhibitory effects of MtRecX, and a partially de-polymerized MtRecA filament could re-polymerize in the presence of MtRecX, which cannot be explained by previous models. Theoretical analysis of force-dependent conformational free energies of naked ssDNA and RecA nucleoprotein filament suggests that mechanical force stabilizes RecA filament, which provides a possible mechanism for the observation. As the antagonizing effect of force on the inhibitory function of RecX takes place in a physiological range; these findings broadly suggest a potential mechanosensitive regulation during homologous recombination.

## INTRODUCTION

In a cell, a variety of factors affect the stability and integrity of the genome resulting in structural damage to the DNA molecule. Homologous recombination (HR) is one of the major pathways that recognizes and repairs the damaged DNA (1). In eubacteria, the RecA protein plays an essential role in HR, by forming a right handed RecA filament on single-stranded DNA (ssDNA) to promote the homologous pairing and exchange of DNA strands in the presence of ATP or ATP-analogues and other co-factors (1,2). The stability of the RecA filament is dynamically regulated by polymerization and de-polymerization in the presence of ATP hydrolysis (1–6).

Various proteins are involved in the regulation of the polymerization and de-polymerization of RecA to avoid either insufficient or unlimited formation of the RecA filament. One such protein, single-stranded DNA binding protein (SSB), outcompetes RecA to bind to ssDNA, inhibiting nucleation and polymerization of the RecA filament, both *in vivo* and *in vitro* (2,7–9). It also promotes net RecA de-polymerization in an ATP hydrolysis- and force-dependent manner (9). The inhibitory function of SSB can be antagonized by the RecFOR complex, which involves three proteins RecF, RecO and RecR (2,10). Besides SSB, the RecX protein strongly inhibits RecA filament nucleation and polymerization, though much less is understood about the RecX protein's regulatory mechanisms.

At substoichiometric concentrations, *Escherichia coli* (Ec) RecX promotes net disassembly of EcRecA filaments on circular DNA in an ATP-hydrolysis-dependent manner, which was explained by a 3′ capping model where EcRecX blocks the growing end (3′ end) of the EcRecA filament, resulting in net EcRecA disassembly (11). More recent experiments showed that higher EcRecX concentrations led to quicker de-polymerization of EcRecA filament (12). In addition, electron microscopic and X-ray crystallographic studies suggested that EcRecX binds deep within the major helical groove in the monomer–monomer interface along the length of the active RecA–ssDNA filament (12–14). Hence, besides the 3′ capping model, an additional internal-nicking mechanism, which produces nicks (therefore more de-polymerization ends) inside the EcRecA filament may exist to facilitate the inhibitory action of RecX, was suggested (12). Finally, it has also been proposed that RecX may facilitate RecA filament end de-polymerization, which is consistent with the observation of RecX concentration dependent RecA filament de-polymerization (15). While aforementioned studies have been focused on RecX promoted RecA de-polymerization, fewer attentions have been paid to the antagonizing factors of RecX inhibitory effects.

Mechanical force has been increasingly recognized as a critical functional factor in diverse biological processes. *In vivo*, forces are produced by various active cellular machineries. For example, a single DNA polymerase or RNA polymerase can exert up to 30 pN on DNA during actions (16,17). Forces are also produced passively by protein-mediated DNA folding. In both eukaryotic and prokaryotic cells, chromosome has regions that are physically attached to the cell wall/membrane (18). Therefore, tension on DNA might increase due to DNA folding by proteins. As protein–DNA interaction typically involves a binding energy of several *k*_B_*T* and nm range interaction distance, forces of a few pN are expected on DNA due to protein binding. Forces in this range have been shown to have major impacts on cell adhesion and migration (19,20) and gene regulation (21). However, the potential effects of these actively or passively produced forces on DNA damage repair have largely remained unexplored.

Recently, an *in vivo* dynamic imaging experiment has shown that during homologous recombinational repair of double-stranded DNA breaks, the two broken ends remained in close proximity while they were moving over large distance during the homologous search process (22). This result indicates that the DNA ends are physically tethered during the whole process and are likely subject to tension. It has been known that the rigid RecA nucleoprotein filament and the soft ssDNA have markedly different force responses (23–25); therefore the stability of RecA nucleoprotein filament is anticipated to be force dependent. However, it has remained unclear regarding the effect of force and its interplay with other cellular factors on the regulation of RecA filament.

In this work we aimed to understand the effect of force on the RecX-mediated regulation of RecA filament stability. We employed single-molecule manipulation technology to directly observe and quantify *Mycobacterium tuberculosis* (Mt) RecX-mediated MtRecA filament dynamics under the regulation of mechanical forces.

## MATERIALS AND METHODS

In the experiments, an in-house built magnetic tweezers setup (26) was used to apply forces on tethered DNA (Supplementary Figure S1). Individual 576 bp dsDNAs were tethered between coverslip and a 2.8-μm-diameter paramagnetic bead (Invitrogen Dynal M280) surfaces at each end of the same strand. The extension change of DNA was recorded at a nanometer resolution with an acquisition frequency of 100 Hz. By dissociating the un-tethered ssDNA strand from the tethered duplex via force-induced DNA strand-peeling transition at ∼65 pN, individual tethered 576 nt ssDNA can be obtained (27–29). The details of the DNA constructs, DNA extension measurements and force measurements are described in our previous publication (9) and Supplementary Methods S1. MtRecX and MtRecA proteins were purified as described (30,31). The standard assay buffered solution containing 20 mM Tris (pH 7.4), 50 mM KCl, 10 mM MgCl_2_, 1 mM ATP and 1x ATP regenerating system (note that 1 mM ATP and 1x ATP regenerating system were included to maintain 1 mM ATP in solution. In the main text and figures, it is referred as 1 mM ATP for simplicity). All experiments were done at 23°C. Refer to Supplementary information for more details of step finding algorithm, kinetics simulation and supplementary figures.

## RESULTS

### MtRecA effects on the force responses of ssDNA

**Figure 1.**
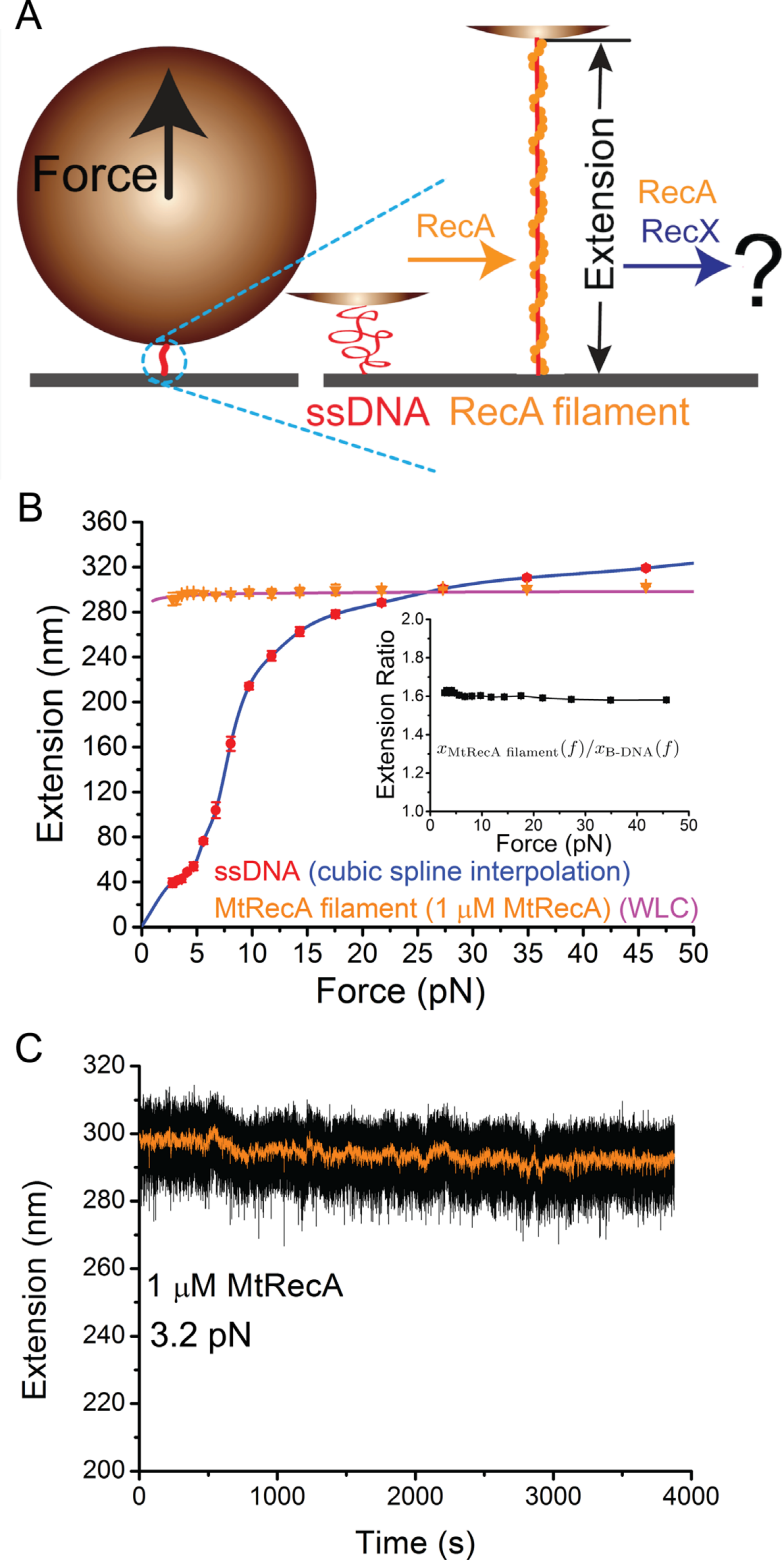
Force response and stability of MtRecA filament. (**A**) Schematic of the experiment. A short ssDNA tethered between a cover glass surface and a paramagnetic bead is subject to forces applied to the bead in the upward direction (left panel). Middle and right panels show a coiled ssDNA (red), and a rigid, extended RecA filament (orange). The effects of MtRecX were explored by the changes in extension of preformed MtRecA filament after induction of MtRecX. (**B**) Typical force-extension curves of a naked 576 nt ssDNA (red circles: experimental data; blue line: cubic spline interpolation of data) in the standard assay buffer and a fully formed MtRecA filament (orange tri-angles: experimental data; magenta line: WLC fitting) on the same ssDNA with 1 μM MtRecA in solution. Inset shows the measured extension of fully formed MtRecA filament divided by the theoretical extension of a B-form dsDNA of equal number of base pairs (576 bp). The error bars are standard deviations (s.d.) obtained from repeating measurements (>3 times) of the same DNA tether under each condition. (**C**) Long time trace of the extension of a preformed MtRecA filament (the same filament as in (B)) recorded at ∼3.2 pN with 1 μM MtRecA in solution.

ssDNA is a very flexible polymer, with an estimated contour length (∼0.6–0.7 nm/nucleotide (nt)) and a small Kuhn length (∼1.5 nm, corresponding to ∼0.75 nm bending persistence length, in 150 mM NaCl (32)). In contrast, a fully formed RecA filament is a very rigid polymer, with an estimated contour length (∼0.52 nm/nt) and a very high bending persistence length (∼1000 nm bending persistence length) (24,25). The sharp difference in their bending rigidity results in very different force responses, which can be quantified in single-DNA stretching experiments in the absence and presence of RecA filament, as sketched in Figure 1A.

Figure 1B shows the force-extension curves of a naked 576 nt ssDNA and the same ssDNA after it was fully coated with an MtRecA filament in standard assay buffered solution containing 20 mM Tris (pH 7.4), 50 mM KCl, 10 mM MgCl_2_, 1 mM ATP and 1x ATP regenerating system at 23°C. At each force, the DNA was held for 5 s to obtain the extension. The error bars are standard deviations (s.d.) from multiple (>3) stretch (force-increasing)-relax (force-decreasing) cycles obtained from the same DNA.

Previous studies have shown that the extension of naked ssDNA as a function of force, $x_{{\rm ssDNA}} (f)$, in monovalent salt solution can be fitted with freely-joint-chain (FJC) polymer model (32) or by a phenomenological polymer model that takes into consideration of the monovalent salt concentration (33). However, neither of these models can fit the force-extension curves of ssDNA measured in MgCl_2_ solution (Supplementary Figure S2), which is likely due to the MgCl_2_ mediated ssDNA condensation through stabilizing the secondary structures formed at low forces (9,34). In our solution, the force-extension curve of the naked ssDNA over the whole force range can be obtained by cubic spline interpolation (Figure 1B, blue line). The force-extension curve of the MtRecA filament, $x_{{\rm RecA}} (f)$, can be fitted by the worm-like-chain (WLC) polymer model using the Marko-Siggia formula (35): $x_{{\rm RecA}} (f) = Nl\left( {1 - \sqrt {k_{\rm B} T/(4Af)} } \right)$, where *l* = 0.52 nm and *A* = 1000 nm are the contour length per nt and the bending stiffness of the MtRecA–ssDNA filaments (Figure 1B, magenta line). The fitted force-extension curves of ssDNA and RecA filament are used in the subsequent section for discussions of the effects of force on regulation of RecA filament stability.

For forces below 30 pN, the extension of MtRecA filament is always longer than the extension of naked ssDNA (Figure 1B). Additionally, the extension difference is larger at smaller forces, as at low forces the flexible ssDNA coils, while the more rigid MtRecA filament remains extended. At forces <7 pN, MtRecA filament extension is at least three times longer than naked ssDNA extension. Furthermore, the extension per nucleotide of the MtRecA filament is 1.5–1.6 times of that of double-stranded DNA per base pair in the force range of 0–50 pN (Figure 1B, inset). These observations are consistent with those from multiple independent experiments on different ssDNA tethers (Supplementary Figure S3). In addition, preformed MtRecA filaments are stable with 1 μM MtRecA in standard solution over a wide force range – no significant net de-polymerization occurred over a long period of time (> 1000 sec) in 1–90 pN force range (Figure 1C, Supplementary Figure S4).

### MtRecX catalyzes ATP-hydrolysis-dependent net stepwise MtRecA filament de-polymerization at low forces

**Figure 2.**
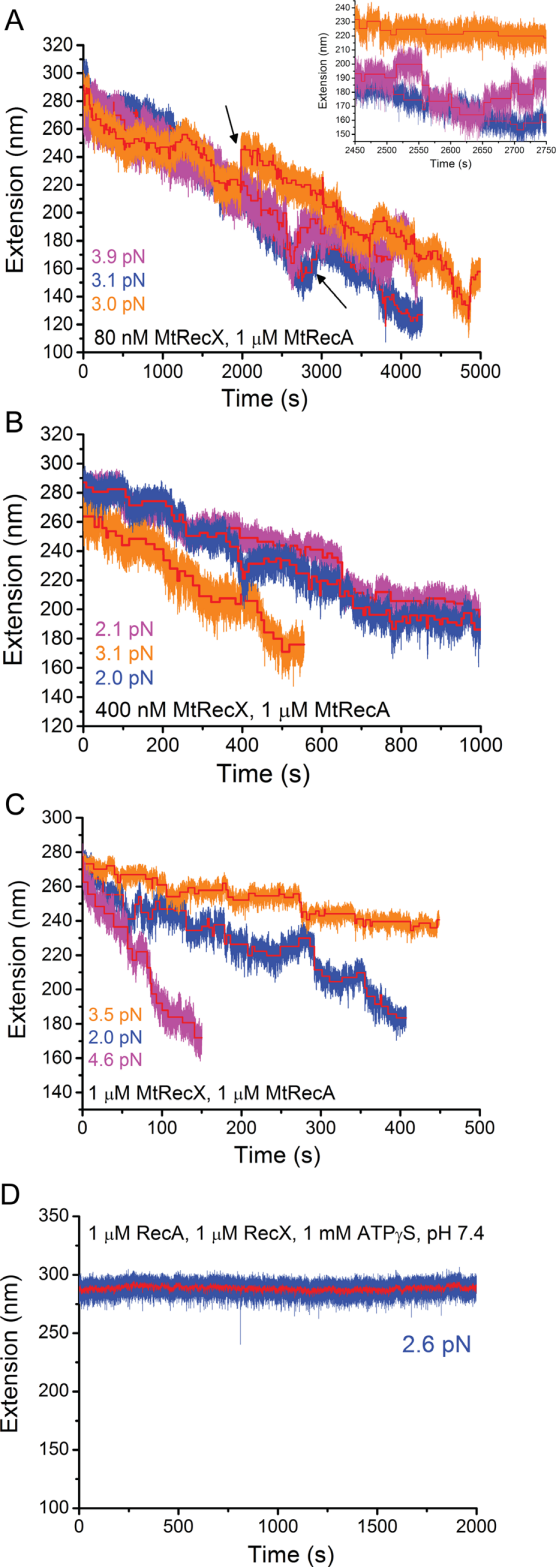
De-polymerization of preformed MtRecA filaments at different MtRecX concentrations. (**A**)**.** Time traces of the extension obtained on three independent preformed MtRecA filaments formed on three ssDNA tethers (indicated by different colors) after addition of 80 nM MtRecX at forces of 2 - 4 pN. Inset shows steps in zoom-in time traces. (**B**) and (**C**) show extension time traces of three independent preformed MtRecA filaments at 400 nM MtRecX (B) and 1 μM MtRecX (C) at forces of 2–4 pN. The red lines in (A)–(C) show stepwise de-polymerization and re-polymerization obtained from a step finding algorithm (Method S3). (**D**) A long extension time trace of a preformed MtRecA in 1 μM MtRecX, 1 μM MtRecA, 1 mM ATPγS (other conditions remained the same), where no net de-polymerization of MtRecA filament occurs over the experimental time scale.

To determine how a preformed MtRecA filament is regulated when MtRecX is added to the MtRecA reaction solution, we investigated the effects of MtRecX on the dynamics of disassembly of preformed MtRecA filaments. At low concentrations of MtRecX (80 nM), we observed progressive reductions in DNA extension at ∼3 pN (Figure 2A), indicating net MtRecA filament disassembly. Although noisy, the disassembly process appeared stepwise overall. In addition to the dominant disassembly process, occasionally large step (> 10 nm) MtRecA re-assembly events were also observed (arrows, Figure 2A).

At a higher concentration of MtRecX (400 nM or 1 μM), similar stepwise de-polymerization processes were observed, while the large re-assembly steps were absent (Figure 2B–C). The overall speeds of MtRecA net de-polymerization were faster at higher MtRecX concentrations, 0.15 ± 0.075 (s.d.) nm/s for 400 nM and 0.27± 0.31 (s.d.) nm/s for 1 μM, than at lower MtRecX concentrations of 80 nM, 0.038 ± 0.019 (s.d.) nm/s. The large s.d. of the overall speeds for each concentration (Figure 2A–C, Supplementary Figure S5) suggests that the net de-polymerization of MtRecA is a highly stochastic process.

Importantly, we found that de-polymerization of MtRecA filament by MtRecX is dependent on ATP hydrolysis: when ATP was replaced by its non-hydrolysable homologue, ATPγS, inhibition of MtRecA de-polymerization was sustained over a long period of time (>2000 s) (Figure 2D). In addition, we found that in the presence of 1 μM MtRecX and 1 μM MtRecA, a lower pH (6.1) significantly slowed down (0.053 ± 0.015 (s.d.) nm/s) the overall de-polymerization speed of MtRecA filament compared to speeds at a higher pH (7.4), (0.27 ± 0.31 (s.d.) nm/s) (Supplementary Figure S6). As lower pH stabilizes RecA filaments (10), these results suggest that the activities of MtRecX on de-polymerization of MtRecA filament are likely regulated by environmental factors such as pH that modulate MtRecA filament stability.

In addition, in multiple, independent experiments using the same MtRecX concentrations, different speeds of de-polymerizations were observed (Figure 2A–C). Though there are several possible explanations for this variation, we speculate that it is due to the stochastic nature of the de-polymerization and re-polymerization processes from a single de-polymerizing end, which is supported by kinetics simulations (see details in the Discussion section).

### Force-assisted re-polymerization of MtRecA filament in the presence of MtRecX

In previous sections, we have quantified the MtRecX promoted MtRecA filament de-polymerization at single molecular level under low force range (∼2–3 pN). At the low-force range, ATP-hydrolysis-dependent MtRecA filament de-polymerization mediated by MtRecX is overall consistent with previous reported biochemistry bulk experimental results obtained from RecX activities across several bacterial species (11,31,36). In this section, the potential effects of force on the inhibitory activity of MtRecX on the RecA filament formation and stability are explored.

**Figure 3.**
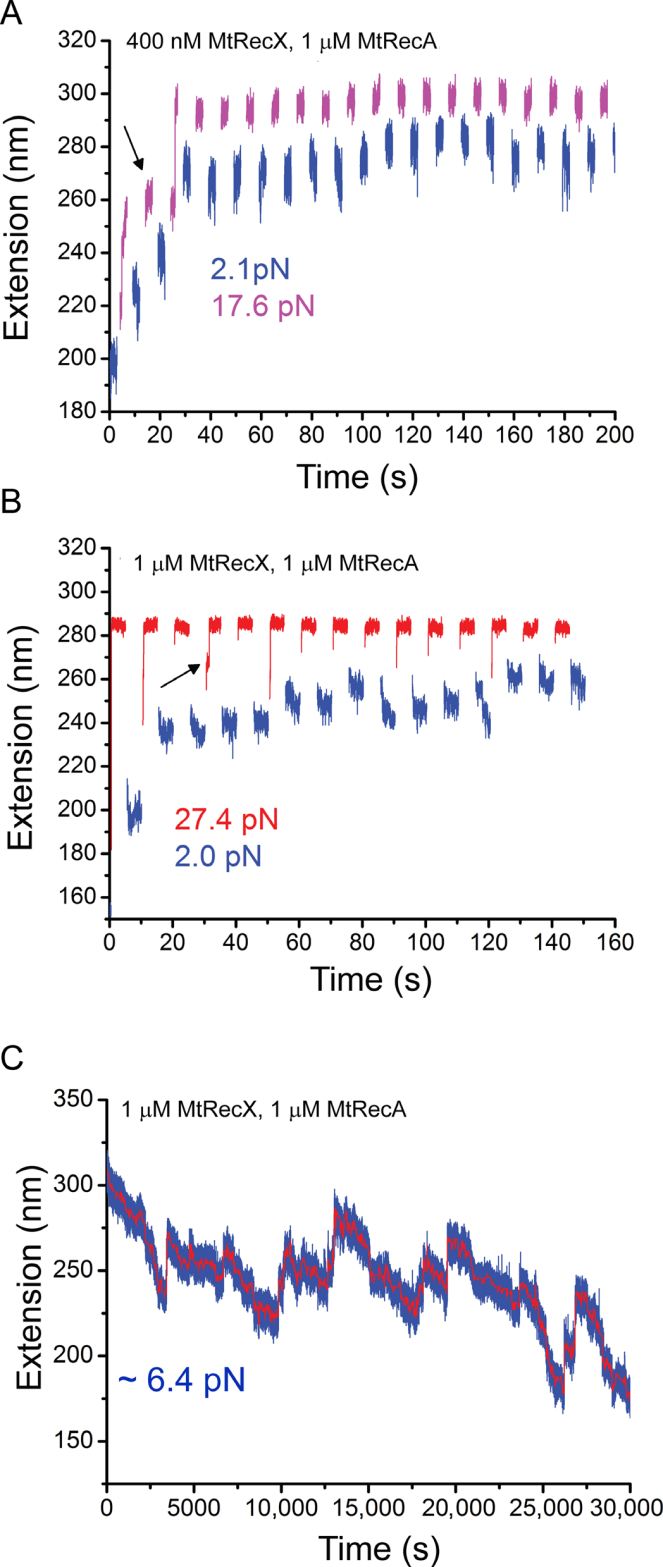
Force assisted re-polymerization of MtRecA filament in the presence of MtRecX. (**A**) Extension evolution of a partially de-polymerized MtRecA filament (the same tether as that indicated by blue color in Figure 2B) during quick jumps between two forces of ∼17.6 pN and ∼2.0 pN with 400 nM MtRecX, 1 μM MtRecA and 1 mM ATP. (**B**) Extension evolution of a partially de-polymerized MtRecA filament (the same tether as that indicated by blue in Figure 2C) during quick jumps between two forces of ∼27.4 pN and ∼2.0 pN with 1 μM MtRecX, 1 μM MtRecA and 1 mM ATP. In both A and B the partially de-polymerized MtRecA filaments re-polymerized at higher forces (>17 pN) after several force-jump cycles, indicated by the elongated extension when force was jump back to lower forces. Black arrows indicate ‘locked’ conformation of MtRecA filament with shorter extension. (**C**) A long extension time trace obtained at ∼7 pN with 1 μM MtRecX, 1 μM MtRecA and 1 mM ATP, showing nearly balanced de-polymerization and re-polymerization over 30,000 s.

To investigate the potential role of force on regulation of the MtRecX-mediated dynamics of MtRecA filament, we monitored the evolution of the extension of partially de-polymerized MtRecA filaments during quick jumps between higher (> 17 pN) and lower forces (∼2.0 pN) (Figure 3A–B). The DNA tether was held for 5 s at each force. After several force-jump cycles, the DNA extension returned to the level of when it is coated with fully polymerized MtRecA filament. This result indicates that the partially de-polymerized MtRecA filament is able to re-polymerize at higher forces even in the presence of 1 μM MtRecX. Similar results were reproduced in multiple experiments and for multiple MtRecX concentrations (80 nM–1 μM) (Supplementary Figure S7). Furthermore, the critical force at which the de-polymerization and re-polymerization of the MtRecA filament are nearly balanced is determined at ∼7 pN (Figure 3C). In addition, we found that MtRecX also catalyzes de-polymerization of EcRecA at lower forces of ∼3 pN (Supplementary Figure S8A), which can also be reversed by applying higher forces (Supplementary Figure S8B). These results suggest that the observed antagonizing effect of force on the inhibitory effects of RecX on RecA filaments is conserved across bacterial species.

Additionally, we observed that in the first few cycles of the force-jump procedure (arrows in Figure 3A and B) the partially de-polymerized MtRecA filament was locked in a mechanically stable short extension state that could withstand large force for several seconds. This locked conformation was unfolded after three or more cycles, indicated by a sudden extension jump. Unfolding of such locked conformations was also observed in several independent experiments (Supplementary Figure S7). While the nature of the locked conformation is unclear, we suspect that the naked ssDNA region was bridged with the partially de-polymerized MtRecA filament region through the secondary binding sites on the MtRecA filament (2), resulting in the locked conformation. In addition, the locked conformation may also involve possible binding of MtRecX to vacated ssDNA. Indeed, at > 500 nM MtRecX concentration, we observed that MtRecX could mediate ssDNA folding, which could be unfolded at > 10 pN force (Supplementary Figure S9).

### Force-dependent de-polymerization and re-polymerization of RecA filament

The above-discovered force-dependent re-polymerization of MtRecA in the presence of MtRecX (80 nM–1 μM) is unexpected from the canonical 5′-to-3′ polymerization of RecA and the 3′ capping model of RecX. This is because if the 3′ end of RecA is capped by RecX, there is no growing end for RecA re-polymerization. To understand the force-assisted re-polymerization, mechanosensitive factors have to be considered.

The stability of RecA filament results from the competition between RecA polymerization and de-polymerization. When a tensile force *f* is applied to the ssDNA, the probability ratio of RecA polymerization and de-polymerization depends on the total free energy cost of polymerization, $\Delta G(f,\xi )$, by the Boltzmann distribution: $p_{{\rm on}} /p_{{\rm off}} = {\rm Exp}( - \Delta G(f,\xi )/k_{\rm B} {\rm T})$, where a parameter vector *ξ* describes the solution conditions such as temperature, salt concentration and pH. $\Delta G(f,\xi )$ consists of a force-independent term, $\Delta G_0 (\xi )$, from the physical interaction between RecA and ssDNA, and a force-dependent term, $\Delta \Phi (f,\xi )$, which is the conformational free energy difference between RecA filament and ssDNA: $\Delta \Phi (f,\xi ) = \Phi _{{\rm RecA}} (f,\xi ) - \Phi _{{\rm ssDNA}} (f,\xi )$. Therefore, at forces when $\Delta \Phi (f,\xi ) {<} 0$, RecA filament formation is promoted by the decreased conformational free energy during polymerization.

**Figure 4.**
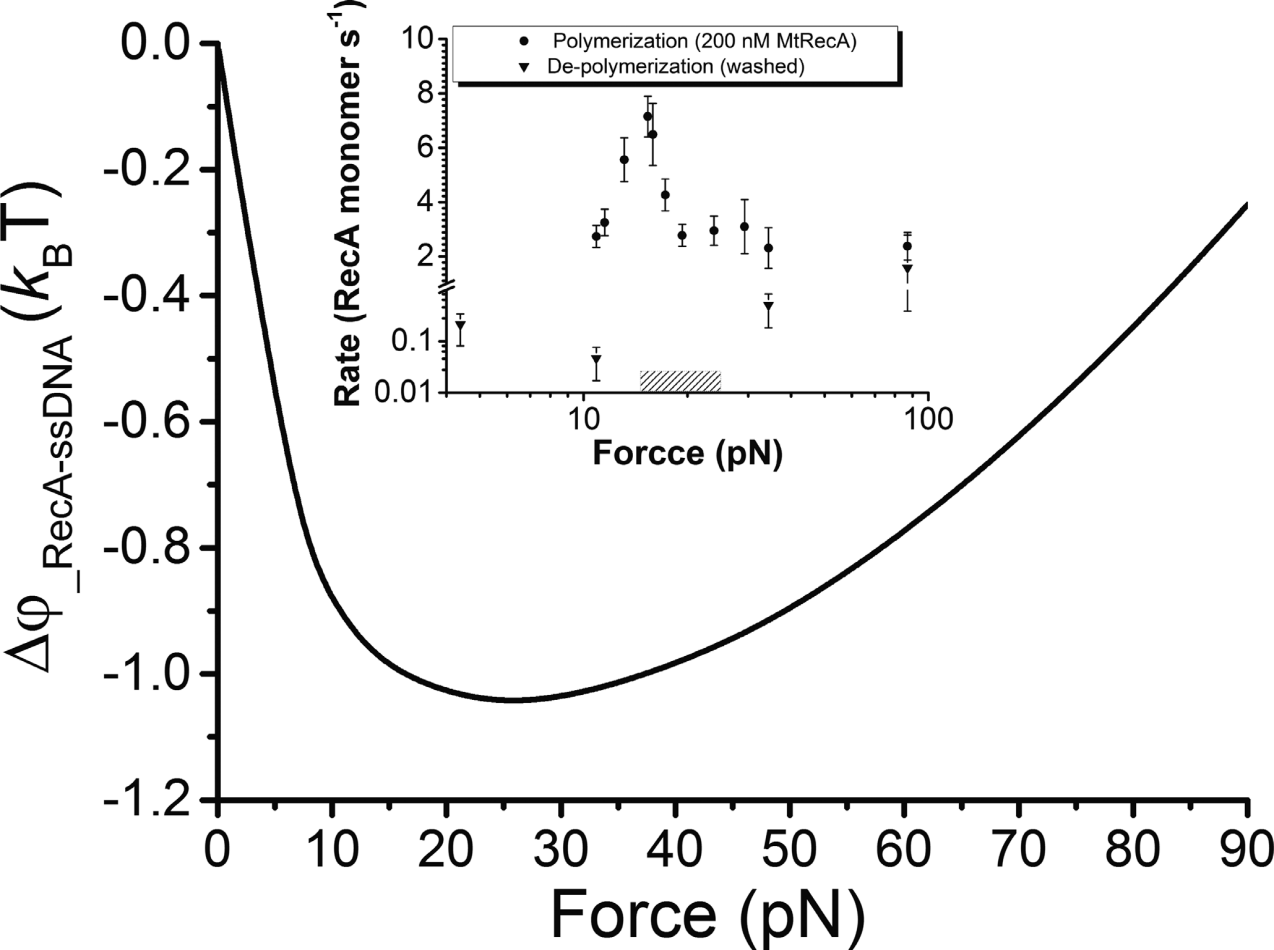
Force-dependent conformational free energy difference between RecA filament and ssDNA. The force-dependent conformational free energy difference per nucleotide between RecA filament and ssDNA, $\Delta \Phi (f) = \Phi _{{\rm RecA}} (f) - \Phi _{{\rm ssDNA}} (f) = \int_0^f {(x_{{\rm RecA}} - x_{{\rm ssDNA}} )df\prime }$, is negative in the force range of 0–90 pN and shows a non-monotonic force dependence. $x_{{\rm RecA}} (f)$ is obtained by WLC fitting and $x_{{\rm ssDNA}} (f)$ is obtained by cubic spline interpolation to experimental data of RecA filament and naked ssDNA in Figure 1B, respectively. Insert shows the experimentally measured polymerization (circles) and de-polymerization (tri-angles) rates of MtRecA filament at multiple forces. Shadow indicates the force range at which no observable de-polymerization of the MtRecA filaments for > 1000 s (Supplementary Figure S10). Note that the polymerization rate was not measured for force <10 pN to avoid formation of secondary structure that may hamper the polymerization of MtRecA on ssDNA.

The force-dependent conformational free energies of RecA filament and ssDNA can be calculated from their respective force-extension curves $x_{{\rm RecA}} (f,\xi )$ and $x_{{\rm ssDNA}} (f,\xi )$ through relations: $\Phi _{{\rm RecA}} (f,\xi ) = - \int_0^f {x_{{\rm RecA}} (f\prime ,\xi )} df\prime$ and $\Phi _{{\rm ssDNA}} (f,\xi ) = - \int_0^f {x_{{\rm ssDNA}} (f\prime ,\xi )df\prime }$. $x_{{\rm RecA}} (f,\xi )\;{\rm and}\;x_{{\rm ssDNA}} (f,\xi )$ were directly measured in our buffered reaction solution conditions (Figure 1B), which were used to calculate $\Delta \Phi (f,\xi )$ per nucleotide ssDNA. As $\Delta \Phi (f)$ is negative up to 90 pN, force in this range facilitates RecA polymerization by reducing the free energy cost for polymerization and is optimized in force range of 20–25 pN (Figure 4). The equilibrium between RecA polymerization and de-polymerization depends on the rates of polymerization and de-polymerization, *k*_on_ and *k*_off_, respectively, through $p_{{\rm on}} /p_{{\rm off}} = k_{{\rm on}} /k_{{\rm off}}$. Therefore, the effect of force on shifting the equilibrium must be through either increasing *k*_on_ or decreasing *k*_off_, or both.

To investigate the effects of force on the on-rate, we recorded the extension change of the ssDNA right after 200 nM MtRecA was introduced at different forces in 50 mM KCl, 10 mM MgCl_2_, 20 mM Tris (pH 7.4), with 1 mM ATP, at 23°C. Similarly, the force-dependent off-rate was measured for a fully polymerized MtRecA filament after removal of free RecA in the channel at different forces (Supplementary Figure S10). Our results show that force facilitates polymerization while it suppresses de-polymerization, with maximal effects around 20 pN (Figure 4, inset).

These non-monotonic trends of the force dependence of polymerization and de-polymerization rates are in general consistent with our theoretical analysis based on the force-dependent conformational free energy difference between RecA filament and ssDNA. We note that *k*_off_ likely indicates the de-polymerization rate of a single de-polymerization end since it began with a fully polymerized filament and there are no free RecA in solution; while *k*_on_ may involve multiple polymerization ends since MtRecA may nucleate at any position on the DNA. In addition, *k*_on_ determined this way is the outcome of competition between RecA polymerization and de-polymerization at this condition.

## DISCUSSION

**Figure 5.**
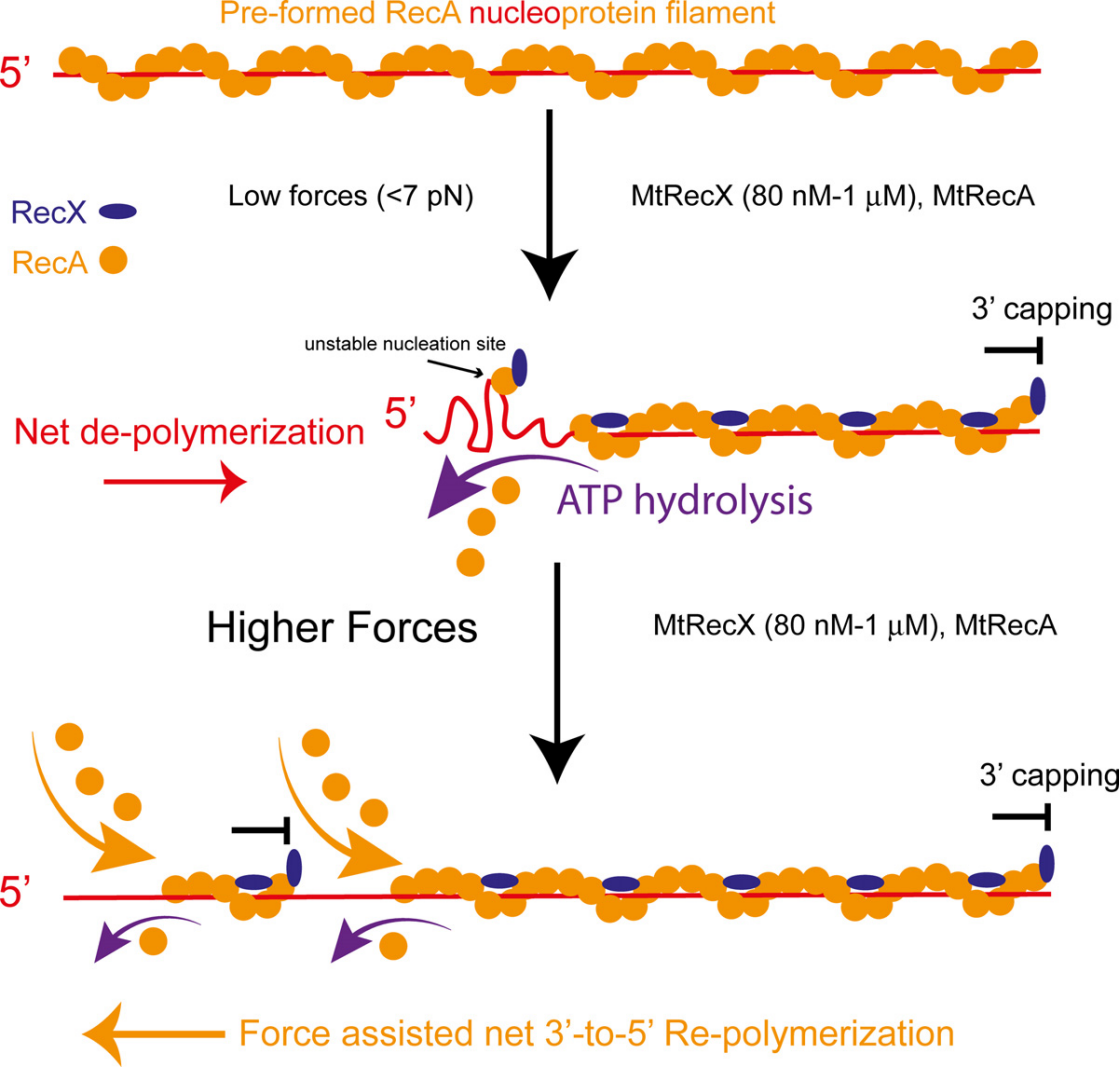
Mechanistic model of the effects of force on MtRecX-dependent MtRecA filament dynamics. A preformed MtRecA filament (orange) on ssDNA (red) is capped by MtRecX (blue) at its 3′ end (based on the 3′ capping model proposed by Dress *et al.* (11)) and is bound with MtRecX at the filament grooves (based on electronic microscopy reconstruction (14)). At low forces (<7 pN according to our measurement), MtRecA dissociates from the ssDNA at the 5′ end that requires ATP hydrolysis, resulting in net de-polymerization. Any potential new nucleation sites formed on the vacated ssDNA are not stable since MtRecX caps them at the 3′ end. At higher forces (>7 pN), due to the stabilizing effect of force on RecA filament, a partially de-polymerized MtRecA filament may re-polymerize from the 5′ end of the remained filament and/or from the new nucleation sites in a force assisted 3′-to-5′ reverse direction.

In this study, we show that at low forces (<7 pN) MtRecX promotes stepwise net de-polymerization of MtRecA filaments in an ATP hydrolysis- and MtRecX concentration-dependent manner, which is consistent with previous ensemble experiments that demonstrated net RecA disassembly by RecX (11,13,31). Compared with those early observations, our studies directly probed the dynamics of the MtRecX induced de-polymerization of MtRecA at a single-molecule level. Moreover, we discovered that at higher forces, the de-polymerized MtRecA filament is able to re-polymerize (Figure 5). This force-assisted re-polymerization of MtRecA in the presence of MtRecX has never been reported and cannot be explained by previous models. Hence, our results have revealed important new insights into how environments such as force may regulate the activities of RecX on RecA filament.

A 3′ end capping mechanism was previously proposed to explain EcRecX induced disassembly of EcRecA filament (11). In addition to inhibiting the polymerization of a RecA filament at the 3′ end, the capping of the 3′ end also inhibits formation of stable nucleation of RecA on vacated ssDNA. The combined effect can explain the net de-polymerization at the 5′ end. Together with previous studies of RecX from *E. coli* and *Neisseria Gonorrhoeae* (11,15), our results suggest that the 3′ capping mechanism is likely a universal dominating factor regulating RecA de-polymerization by RecX across bacterial species.

The 3′ capping mechanism also explains the stochastic variations in overall de-polymerization speeds observed in our experiments. In our assays, de-polymerization began with a fully polymerized MtRecA filament presumably with the 3′ end capped by a single RecX molecule according to this mechanism. This leaves the 5′ end as the only end for de-polymerization, which is facilitated by ATP hydrolysis. The position of the 5′ end is determined by two competitive processes: ATP-hydrolysis-dependent de-polymerization and reverse re-polymerization from the 5′ end. As ATP hydrolysis and spontaneous dissociation and association of RecA are stochastic processes (5), the position of the de-polymerizing 5′ end undergoes a one-dimensional random walk along the ssDNA track. Using the experimentally estimated de-polymerization and re-polymerization rates as well as the step sizes (Supplementary Discussion S1, Supplementary Methods S3 and Supplementary Figures S11 and S12), the predicted extension evolution of a preformed MtRecX filament using kinetics simulation algorithm (Methods S4—kinetics simulation) is consistent with the ones observed in experiments (Discussion S2, Supplementary Figure S13).

Importantly, we observed that a partially de-polymerized MtRecA filament is able to re-polymerize at higher forces (>7 pN) in the presence of MtRecX (80 nM to 1 μM). This finding is unexpected because it seems to suggest that the 3′ capping mechanism does not apply to MtRecA filaments under sufficiently large force. To explain this force-assisted re-polymerization, some mechanosensitive factors must be considered.

One possibility is that force suppresses ATP-hydrolysis-dependent de-polymerization rate and polymerization rate at the 5′ end, shifting the balance toward the re-polymerization. In this scenario, re-nucleation and 5′-to-3′ re-polymerization of MtRecA take place on naked ssDNA vacated during de-polymerization, assisted by force. As any newly nucleated MtRecA segments should be quickly capped by MtRecX at their 3′ ends, this picture implies multiple de-polymerization ends of the re-polymerized MtRecA and predicts a quicker de-polymerization rate upon switching to a low force. However, the de-polymerization kinetics observed in our experiments can be explained by stochastic de-polymerization of a single de-polymerization end; therefore, this mechanism alone is not directly supported by our results.

Another possibility is a potential force-assisted reversed (3′-to-5′) re-polymerization of the partially de-polymerized MtRecA filament. In this scenario, although MtRecX blocks the 3′ end, the 5′ end of the filament may still extend in a reverse direction under conditions that the 3′-to-5′ re-polymerization rate outcompetes the de-polymerization rate (see sketch in Figure 5). While a canonical 5′-to-3′ polymerization of RecA has been widely known, the existence of reversed 3′-to-5′ polymerization has recently been demonstrated by several groups for EcRecA (3,7,9,37). In the supplementary information we also provided evidences for reversed 3′-to-5′ polymerization of MtRecA on MtSSB coated ssDNA assisted by force (Discussion S3, Supplementary Figure S14). The force-promoted reverse polymerization of RecA filament may take place from the end of a partially de-polymerized filament and/or from potential new RecA nucleation sites whose 3′ end is capped by RecX on vacated ssDNA. Besides the above-discussed force assisted reverse polymerization, we note that the canonical 5′-to-3′ polymerization might also take place from new nucleation sites on vacated ssDNA if the 3′ capping capability of MtRecX is reduced by mechanical force.

Based on the distinct force-extension curves of naked ssDNA and RecA filament, our calculation has shown that forces up to 90 pN decrease the free energy cost of RecA polymerization, with a maximal effect in ∼20–25 pN. This is purely a mechanical effect that stabilizes RecA filament in certain force range. Our polymerization and de-polymerization experiments in the absence of RecX have shown strong non-monotonic force dependence of both the polymerization and de-polymerization rates, which are generally consistent with the theoretical predicted trends. We note that in a previous study, it was reported that the de-polymerization rate of EcRecA filament monotonically decreases as force increases up to 16 pN (38), which does not contradict to our results due to the lower force range used in that experiment. Due to the above-discussed stabilizing effect of mechanical force, we reason that a partially de-polymerized MtRecA filament in the presence of MtRecX and MtRecA in solution could re-polymerize in a reverse direction at sufficiently high force.

In summary, our findings from MtRecX, together with previous results obtained from EcRecX and NgRecX (11,13,15), advance our understanding of RecX function across bacterial species. The importance of mechanical forces is highlighted by its antagonizing the inhibitory effects of MtRecX on MtRecA filament stability. Moreover, as the effect of force mainly comes from the distinct force responses between naked ssDNA and RecA filament, we expect that force may be generally involved in suppressing RecA inhibitory factors such as RecX and SSB, while it may enhance the RecA positive mediating proteins such as RecFOR complex. Overall, these findings underscore a need to further explore the mechanosensitive regulation of homologous search and recombination reactions.

## SUPPLEMENTARY DATA

Supplementary Data are available at NAR Online.

SUPPLEMENTARY DATA
