# Supplementary material for: Mechanical force antagonizes the inhibitory effects of RecX on RecA filament formation in Mycobacterium tuberculosis
Source: Nucleic Acids Res. 2014 Oct 7;42(19):11992–9. doi: 10.1093/nar/gku899 (PMC4231760; doi:10.1093/nar/gku899)
Supplement: SUPPLEMENTARY DATA [file supp_42_19_11992__index.html]

Mechanical force antagonizes the inhibitory effects of RecX on RecA filament formation in Mycobacterium tuberculosis — SUPPLEMENTARY DATA 

# Mechanical force antagonizes the inhibitory effects of RecX on RecA filament formation in *Mycobacterium tuberculosis*

## SUPPLEMENTARY DATA

**Files in this Data Supplement:**

- SUPPLEMENTARY DATA
