# Supplementary material for: Mechanical force antagonizes the inhibitory effects of RecX on RecA filament formation in Mycobacterium tuberculosis
Source: Nucleic Acids Res. 2014 Oct 7;42(19):11992–9. doi: 10.1093/nar/gku899 (PMC4231760; doi:10.1093/nar/gku899)
Supplement: SUPPLEMENTARY DATA [file supp_gku899_nar-01402-f-2014-File007.pdf]

Supplementary information for

Mechanical force antagonizes the inhibitory effects of RecX on RecA filaments formation in *M. tuberculosis*

SI Text—Supplementary Materials, Methods and Discussions

Methods S1 – Magnetic Tweezers setup, DNA and flow channel preparation

In the in-house built magnetic tweezers setup, a pair of permanent magnets used to generate magnetic field gradient (z-direction) perpendicular to the coverslip surface (x-y plane) (Figure S1). Hence, a tethered micro superparamagnetic bead on the coverslip is subject to forces toward the direction of magnetic field gradient. The magnitude of the force is tuned by controlling the distance between the coverslip surface and the magnets. Backscattered illumination using LED light source is used, so the gap between the two magnets can be minimized to maximize the magnetic field gradient. A CCD/CMOS (short for Charge coupled device/complementary metal oxide semiconductor) camera was used to collect the images obtained and transfer to a computer for further imaging analysis. More details of the setup can be found in (1).

The 576 bp dsDNA was produced by PCR from lambda phage DNA with following primers:

5'[Thiol]ATTATACTCGAGAGCATAAGCAGCGCAACA3' (forward primer)

5'ATTATAGAATTCATGACGCAGGCATTATGCT3' (reverse primer).

The dsDNA product was then digested by Hind III enzyme, and labeled with biotin by incubation with Biotin-dUTP and Vent DNA polymerase. Finally, the DNA was labeled with thiol and biotin at the two ends of the same DNA strand.

The flow channel was made with two pieces of coverslips sticking together by parafilms (Figure S1). The bottom coverslips was cleaned and sinalized with (3-Aminopropyl) triethoxysilane (APTES) (Sigma). Then, it was coated with sulfo-SMCC by incubation with sulfo-SMCC (Sigma) in 1x PBS buffer for ~30 minutes.

The thiol and biotin labeled dsDNA in 1x PBS solution was then flowed into the sulfo-SMCC channel, and incubated for ~ 30 min, followed by incubation with 2% BSA in 1x PBS solution for 2 hours for surface blocking. Finally, 1x PBS solution with streptavidin-coated paramagnetic beads (2.8 μm in diameter) was introduced into the channel and incubated for ~ 10 minutes to form DNA tethers.

During the experiments, a single dsDNA tether was identified and melted into an ssDNA by force-induced peeling of the unlabelled strand away during DNA overstretching transition under the conditions where the strand peeling occurs (2-4).

Methods S2—Force-jumping measurement

The extension difference between naked ssDNA and RecA filament are relatively small ([-20, 20] nm) at forces > 15 pN (refered as high force range) (Figure 1B). The small extension difference results in difficulty in monitoring the extension evolution from naked ssDNA to RecA filament (*vice versa*) in the high force range. In contrast, at lower forces (<15 pN) the extension difference is much larger. In order to study the effects of the high force during the RecX mediated RecA filament dynamics, a so-called "force-jumping" measurement was applied: In an experiment, the DNA tether was held a high force (>15 pN) for serveral seconds during which RecA

filamanet may polymerize or depolymerize. In order to check the extent of RecA filament change during this period at the high force, we jumped the force to a lower value (<10 pN) to get the extension at the lower force. Should RecA filament polymerize during the high force interval, the extension at low force is expeced to increase (and *vice versa*). By repeating the force jumping cycles, we could invetigate the dynamics RecA filament polymerization and depolymerization at high forces. Note that the time that it takes jumping one force to another is < 0.1 second.

Methods S3--Step finding algorithm

A step finding algorithm similar to that developed by Cui et al. (5) was used to estimate the steps sizes and kinetics of MtRecA filament dynamics. First, the raw data were smoothed by the Savitzky-Golay method within 0.2 sec time window. Then a local extension difference $\Delta_{0.1s}$ (t), which is the extension measured at time t minus the extension measured at t-0.1s, is calculated throughout the whole time trace, and a global standard deviation, σ, is obtained. Trial steps are detected when $\Delta_{0.1s}$ (t) exceeds 2σ. Typical noise levels in our experiments are in the range of 2σ ~ 2-4 nm for partially de-polymerized MtRecA filaments depending on the amount of vacated flexible ssDNA during de-polymerization. Only steps with the Student's T-value greater than 7 were accepted as de-polymerization or re-polymerization steps, and the corresponding step size and the time point of the accepted stepwise extension changes were recorded. A typical step finding process was plotted as Figure S11.

Methods S4--Kinetics simulation

In the Kinetics simulation, a small time step Δt = 0.01 s (corresponding to the experimental temporal resolution 100 Hz) was chosen, within which the probabilities for de-polymerization, re-polymerization, and neither de-polymerization nor re-polymerization to occur are $p_{off}(\Delta t) = k_{off}\,\Delta t$, $p_{on}(\Delta t) = k_{on}\,\Delta t$, and $p_{none}(\Delta t) = 1-(p_{off}(\Delta t) + p_{on}(\Delta t))$, respectively. A uniformly distributed random number $0 < r < 1$ was generated, which is compared to the above probabilities: de-polymerization is selected if $0< r < p_{off}(\Delta t)$; re-polymerization is selected if $p_{off}(\Delta t) < \mathrm{r} < p_{off}(\Delta t)+ p_{on}(\Delta t)$; and neither de-polymerization nor re-polymerization is selected if $p_{off}(\Delta t) + p_{on}(\Delta t) < r < 1$.

Discussion S1— Kinetics of MtRecX mediated net de-polymerization of preformed MtRecA filaments

To gain further insights into the variations of the net de-polymerization speeds of preformed MtRecA filaments, we analyzed the de-polymerization and re-polymerization steps in each time trace. A step finding algorithm was employed to extract negative steps (de-polymerization) and positive steps (re-polymerization) from the data. Detection of such stepwise signal was automated by detecting abrupt decreases or increases in extension using a method similar to that developed by Cui *et al.* (5) (Methods S3--Step finding algorithm). Red lines in the Figure 2 A-C show the stepwise time traces identified by this algorithm. Note as this method cannot detect steps smaller than the noise level, the numbers of potential steps as well as the transition rates are likely underestimated.

In the absence of MtRecX, ~ 2 nm steps were detected for ATP-dependent

MtRecA dissociation and re-association, which are larger than the expected monomer dissociation and re-association steps. Note a RecA monomer is known to associate three consecutive nucleotides of ssDNA, corresponding to ~ 0.9 – 1.2 nm ssDNA extension changes at 2 - 4 pN per MtRecA monomer dissociation or re-association (Figure S12A). Such small steps are below the noise level (~ 2 nm for smoothed data) of the extension fluctuation; therefore they cannot be detected. The averages, standard deviations, and standard errors of the step sizes and the rates of de-polymerization and re-polymerization are shown in Figure S12B-C.

In the presence of MtRecX in the range of 80 nM – 1 μM and force range of 2 - 4 pN, the average step sizes for both de-polymerization and re-polymerization are around 4 nm, roughly corresponds to release of 9 nt of ssDNA, or equivalently simultaneous dissociation of three RecA monomers (i.e., half helical turn of RecA filament) (Figure S12A). Although the causes of this step wise de-polymerization is unclear, it may be related to cooperative ATP hydrolysis in the RecA filament reported (6). Over the range of MtRecX concentrations, the step sizes do not depend on the MtRecX concentration. In contrast, the kinetics of the de-polymerization is dependent on the MtRecX concentration. From 80 nM to 1 μM MtRecX, the average de-polymerization rate increases by ~ 3-folds, and the ratio of de-polymerization rate over the re-polymerization rate increases by > 2-folds (Figure S12C., inset). These results suggest that MtRecX facilitates the rate of de-polymerization in addition to 3' capping. The large standard deviations suggest a highly stochastic process of de-polymerization.

Discussion S2— Large variations in MtRecA de-polymerization speeds can be explained by stochastic de-polymerization and re-polymerization kinetics

In multiple, independent experiments using the same MtRecX concentrations, we observed different speeds of de-polymerizations (Figure 2A-C). Though there are several possible explanations for this variation, we reason it is likely due to the stochastic nature of the de-polymerization and re-polymerization processes. Each extension time trace can be understood by a one-dimensional random walk process, with an average de-polymerization rate of k_{off} and step size l_{off}, as well as a re-polymerization rate of k_{on} and step size of l_{on}. These parameters were estimated in experiments for each MtRecX concentration (Figure S12). Based on the averages of these parameters, we simulated the extension evolution of a pre-formed MtRecX filament using kinetics simulation algorithm (Methods S3—kinetics simulation).

Using the averaged values of the kinetic and step size parameters estimated, five independent simulated time traces were generated for each corresponding MtRecX concentration over similar time scales (Figure S13). These stepwise time traces were superimposed with Gaussian noise using a standard deviation of ~ 4 nm, which is similar to the noise level of the raw time traces obtained in our experiments. We found that the simulated time traces were consistent with the corresponding experimental time traces. The simulated and experimental traces exhibit similar overall net de-polymerization speeds and large variations from one experiment to another. Hence, the simulation results support our hypothesis that the variations of the extension time traces for multiple, independent experiments using the same MtRecX

concentration can be explained by the stochastic nature of MtRecA de-polymerization and re-polymerization steps of a single filament. RecA polymerization is intrinsically stochastic, as it involves diffusion of free RecA proteins to the filament. De-polymerization is facilitated by ATP hydrolysis mainly taking place at the 5' end. ATP turnover is a stochastic process, and spontaneous dissociation of RecA after ATP hydrolysis should also be a stochastic process. All these may potentially contribute to the stochastic nature of MtRecA de-polymerization process as observed in experiments and simulation. These results do not exclude the possibility that MtRecX may create limited nicks inside the MtRecA filament.

Discussion S3— 5'-to-3' polymerization of MtRecA filament revealed by its re-polymerization on MtSSB bound ssDNA assisted by force

Recently, 3'-to-5' reverse polymerization of RecA in *E.coli* has been demonstrated by several groups (7-10). Here, the possible existence of the 3'-to-5' reverse polymerization of RecA in *M. tuberculosis* was examined. In reaction solution containing 20 mM Tris (pH 7.4), 50 mM KCl, 10 mM $MgCl_2$, 1 mM ATP, at 23 ^oC (the same environmental condition as in main text), an MtSSB protein array was formed on ssDNA in the MtSSB concentration from 0.25 nM to 250 nM. In this concentration range, MtSSB binding resulted in slight extension reduction in a moderate force range of 5-15 pN, which is expected from ssDNA wrapping around MtSSB tetramers. A mixture of 250 nM MtSSB and 1 μM MtRecA was then introduced in the same buffer solution. The force-extension curve remained nearly unchanged, indicating that MtRecA could not nucleate and polymerize on the MtSSB

coated ssDNA under this solution condition (Figure S14A).

When an MtRecA filament was pre-formed on ssDNA followed by introduction of the mixture of 250 nM MtSSB and 1 μM MtRecA, net de-polymerization of the MtRecA filament was observed, indicated by progressive extension reduction at a few pN forces (Figure S14B). This can be explained by binding of MtSSB to vacated ssDNA at the 5' end of the MtRecA filament due to ATP-hydrolysis mediated MtRecA disassociation, resulting in net de-polymerization. Such MtSSB dependent de-polymerization of preformed MtRecA filament should lead to a partition of the ssDNA into MtSSB array at the 5' side of the ssDNA and the remaining MtRecA filament at the 3' side of ssDNA.

Before the MtRecA filament was completely de-polymerized, upon switching to high forces, re-polymerization of the MtRecA filament was observed, revealed by extension re-elongation at low forces. One example is shown in Figure S14C - switching force between a low force (1 pN) and a high force (15 pN) on a partially de-polymerized MtRecA filament resulted in progressive elongation of the ssDNA extension at 1 pN. As this experiment began with a pre-formed fully polymerized MtRecA filament, there was no space left at the 3' end for the canonical 5'-to-3' directional polymerization. The only space on ssDNA available for MtRecA re-polymerization was at the 5' side which was however occupied by MtSSB. Therefore, we conclude that the force-assisted re-polymerization should take place from the 5' end of remained MtRecA filament in a reversed 3'-to-5' direction. Such reversed polymerization was also observed for EcRecA re-polymerization on EcSSB

coated ssDNA at the 5' side (8).

SI Figures

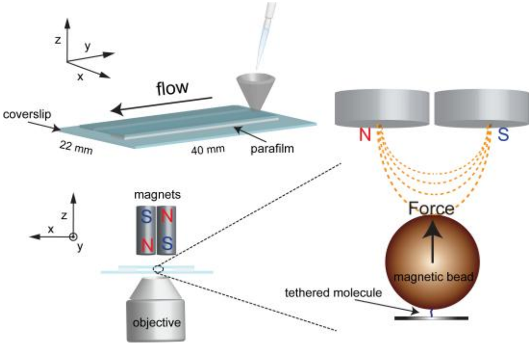

Supplementary Figure S1. Flow channel and basic setup of magnetic tweezers. A flow channel (top left) with a volume of ~ 20 µl is placed on the magnetic tweezers setup (bottom left). The force is applied by a pair of magnets and controlled by the distance between the magnets and the channel surface (right). During experiments, buffered solution with a volume > 200 µl is flowed through the channel for each solution exchange.

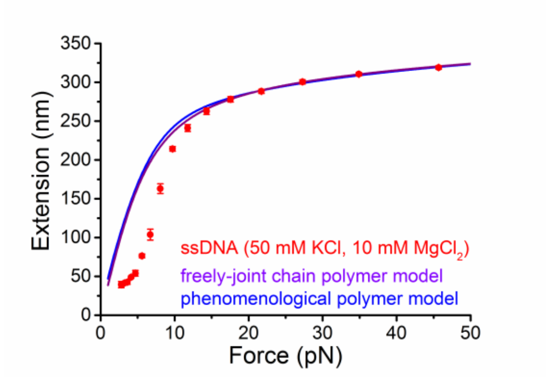

Supplementary Figure S2. Neither freely-joint-chain polymer model nor a phenomenological polymer model can fit the force response of ssDNA in the presence of $MgCl_2$. The solid circles are the experimental data as shown in Figure 1B. The blue line is calculated based on a phenomenological polymer model introduced by Cocoo et al (11): $x_{\text{ssDNA}}(f) = Nh\left(a_1 \frac{\ln\left(\frac{f}{f_1}\right)}{1+a_3 \exp\left(-\frac{f}{f_2}\right)} - a_2 - \frac{f}{f_3}\right)$, with the following

parameters: h=0.34 nm, $a_1 = 0.21$, $a_2 = 0.37$, $a_3 = 2.1\ln(I/0.0025)/\ln(0.15/0.0025) - 0.1$, $f_1 = 0.0037$, $f_2 = 2.9$ pN, and $f_3 = 8000$ pN. Here N is the number of bases and I is the ionic strength which is the molar concentration of monovalent salt. The purple line is calculated based on freely-joint chain model: $x_{\text{ssDNA}}(f) = Nb_{ss}(\coth(\frac{2Af}{k_\text{b}\text{T}}) - \frac{k_\text{b}\text{T}}{2Af})(1 + f/800)$, where $b_{ss} = 0.56$ nm is the contour length of ssDNA per nt, the $A = 0.75$ nm is the persistence of ssDNA (12).

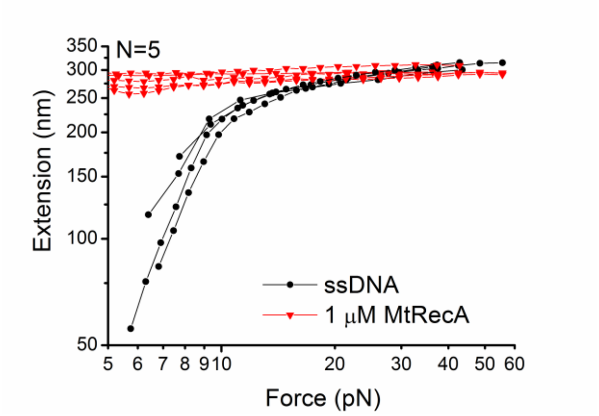

Supplementary Figure S3 Force responses of naked ssDNA and ssDNA bound with MtRecA filaments. Multiple (N=5) independent force responses of ssDNA (black circles) and the MtRecA filaments (red tri-angles) formed on each ssDNA. On naked DNA, at small force range (< 10 pN), the extension variation is likely large due to formation of secondary structure.

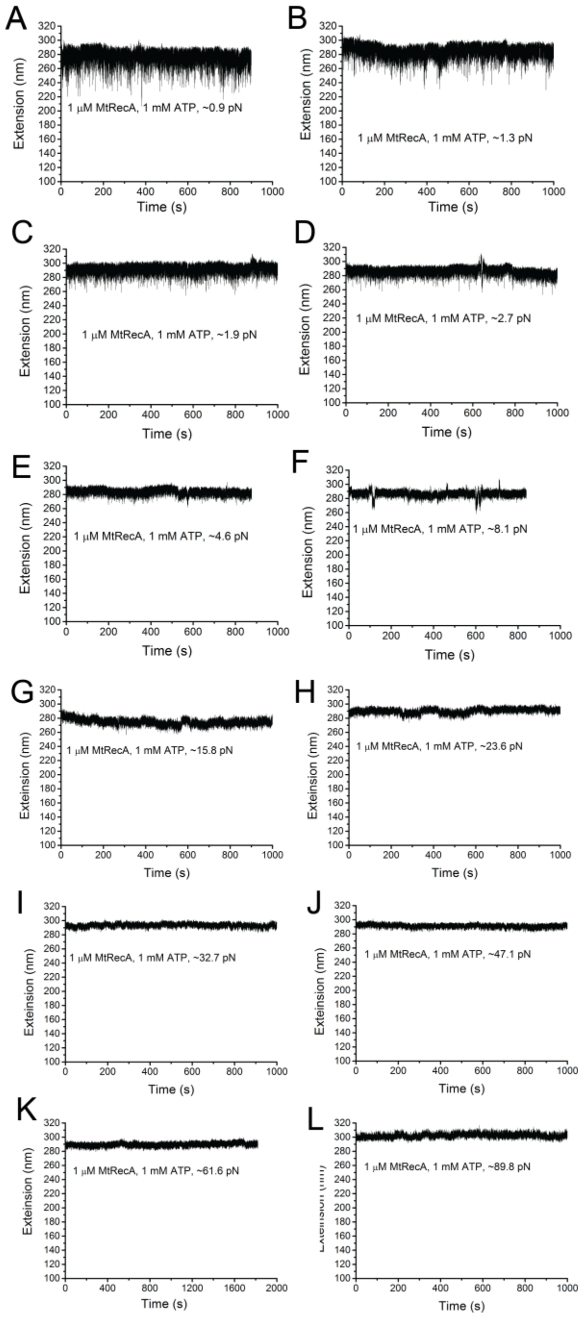

Supplementary Figure S4. MtRecA filament is stable at a wide force range (1-90 pN) in the presence of 1 μM MtRecA in standard reaction solution. (A-L) show the extension time trace of MtRecA filament over 1000 seconds at forces from 1 pN to 90 pN in standard reaction solution with 1 μM MtRecA.

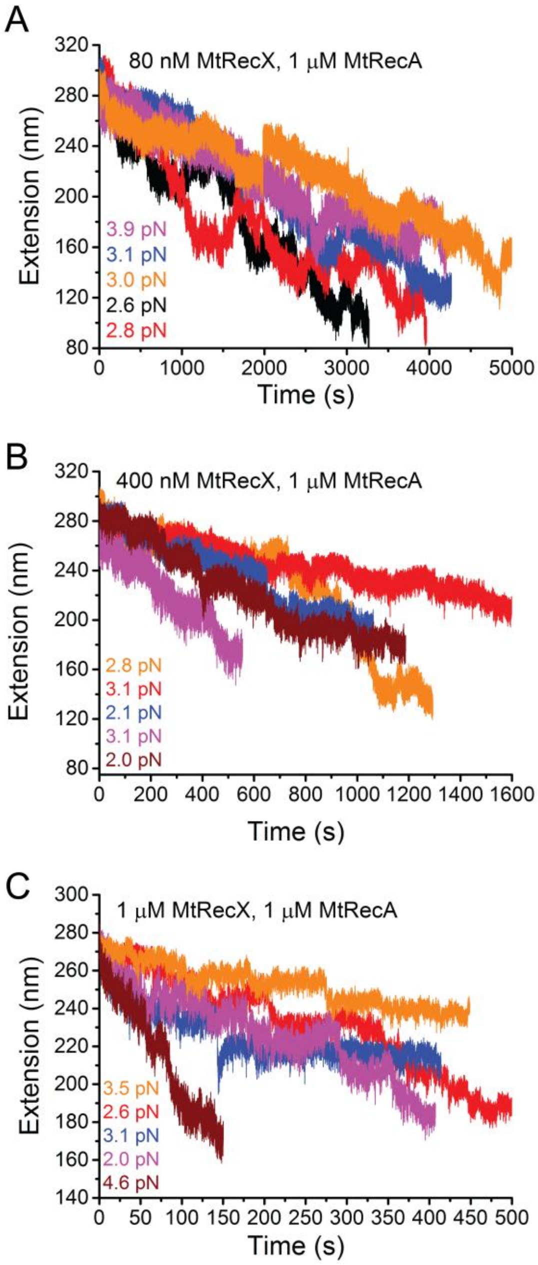

Supplementary Figure S5. Extension time traces of pre-formed MtRecA filament in the solution with 1 μM MtRecA and different concentration of MtRecX (80 nM-1 μM) at low forces of ~ 3 pN. (A-C). At each condition, experiments performed on 5 different ssDNA tethers were recorded. Three of the 5 extension time traces are shown on Figure 2.

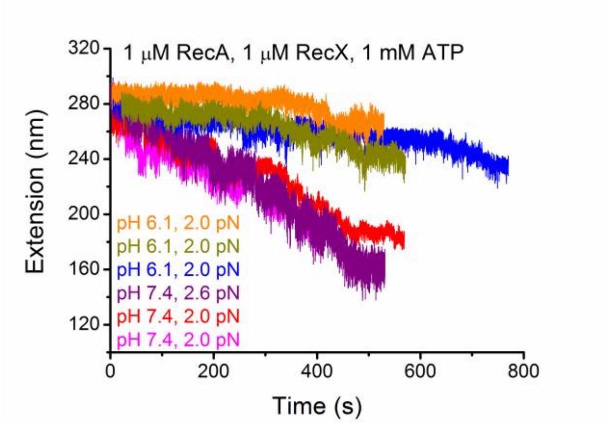

Supplementary Figure S6 Time traces of the extension evolution of preformed MtRecA filament at pH 7.4 or pH 6.1, in 1 μM MtRecX, 1 μM MtRecA, 1 mM ATP, at forces of 2 - 3 pN, which show a much slower de-polymerization speed at lower pH 6.1 (0.053 ± 0.015 nm/s (s.d.)) than at pH 7.4 (0.26 ± 0.27 nm/s (s.d.)). Different time traces have been indicated by different colors.

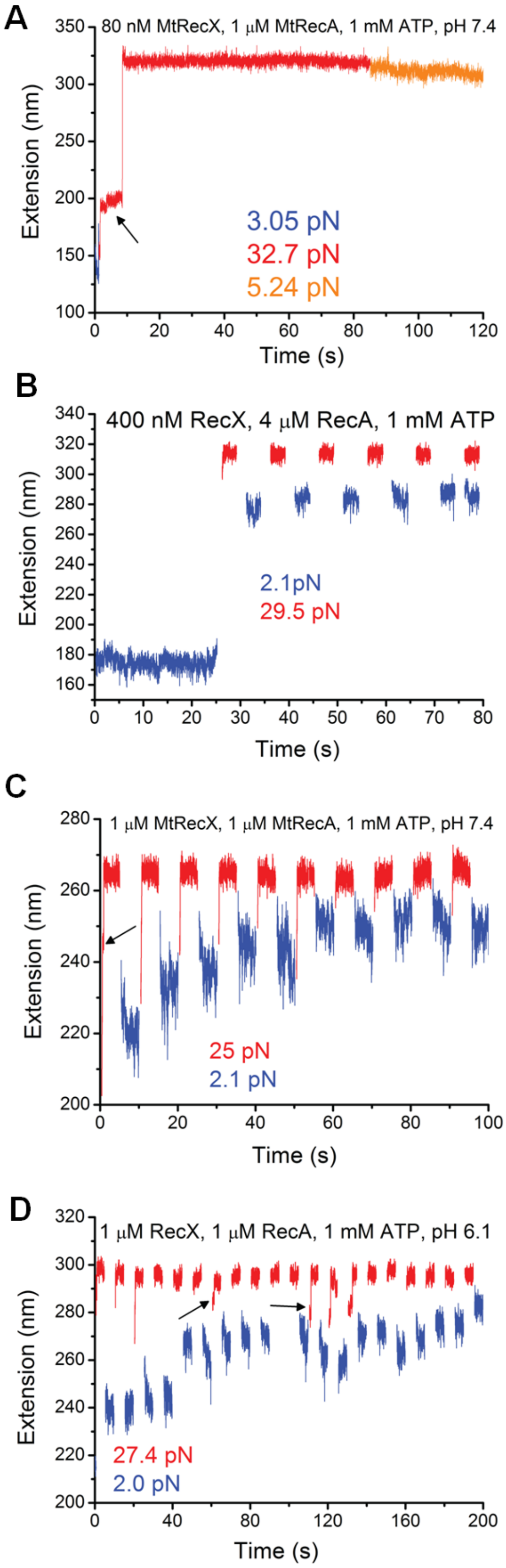

Supplementary Figure S7 Force assisted re-polymerization of MtRecA filament in the presence MtRecX ranging from 80 nM to 1 μM. The extension in each figure

returned to the original extension of fully coated MtRecA filament after several (>3) cycles of force jumping between higher forces and lower forces. The black arrows represent the possible locked state of MtRecA-ssDNA filament with a shorter extension. The experiments were performed in standard reaction solution at 23 ^{o}C.

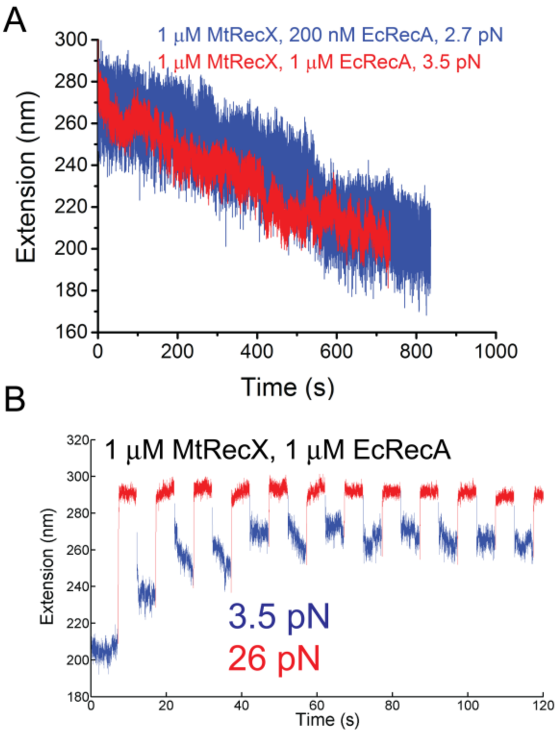

Supplementary Figure S8. Force dependence of MtRecX mediated EcRecA filament dynamics. (A). MtRecX (1 μM) promotes net de-polymerization of EcRecA filaments (200 nM and 1 μM tested) at low forces of ~ 3 pN. (B). Higher forces assist re-polymerizaiton of partically de-polymerized EcRecA filament in the presence of MtRecX.

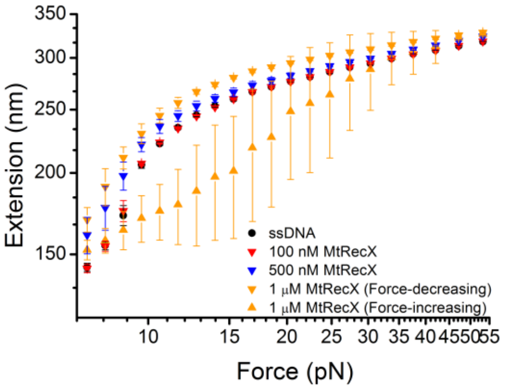

Supplementary Figure S9. Typical force-extension curves of an ssDNA in various concentrations (1 nM-1 μM) of MtRecX. The force-extension curves of ssDNA with MtRecX < 100 nM are essentially overlapped with that of ssDNA, therefore were not shown for simplicity. The error bars are standard deviations (s.d.) obtained from repeating measurements (>3 times) of the same DNA tether under each condition. The force-extension curves of the ssDNA in the presence of 1 μM MtRecX has a large hysteresis between extensions recorded in force-decrease and the following force-increase scans.

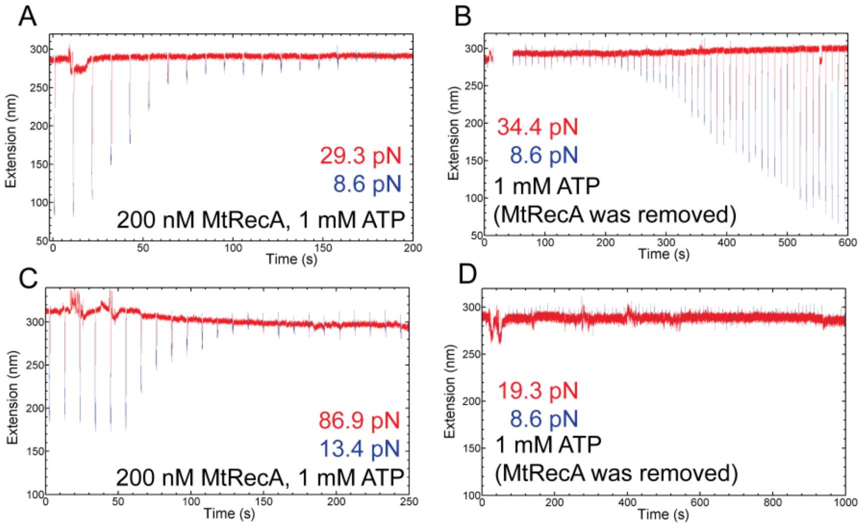

Supplementary Figure S10. Typical RecA filament extension time traces recorded

during force jumping cycles. **(A&C)**. A naked ssDNA was held between a higher force (red) for 10 seconds and a smaller force (blue) for 0.5 seconds. Data in the first 25 seconds were recorded before and during 200 nM MtRecA was introduced into the channel. After 200 nM MtRecA was introduced, the polymerization process of MtRecA filament at higher force was checked by the extension change at the lower force. **(B&D).** Similarly, the de-polymerization process of MtRecA filament at higher forces could also be examined by the extension change at the lower forces after removal the free RecA in solution.

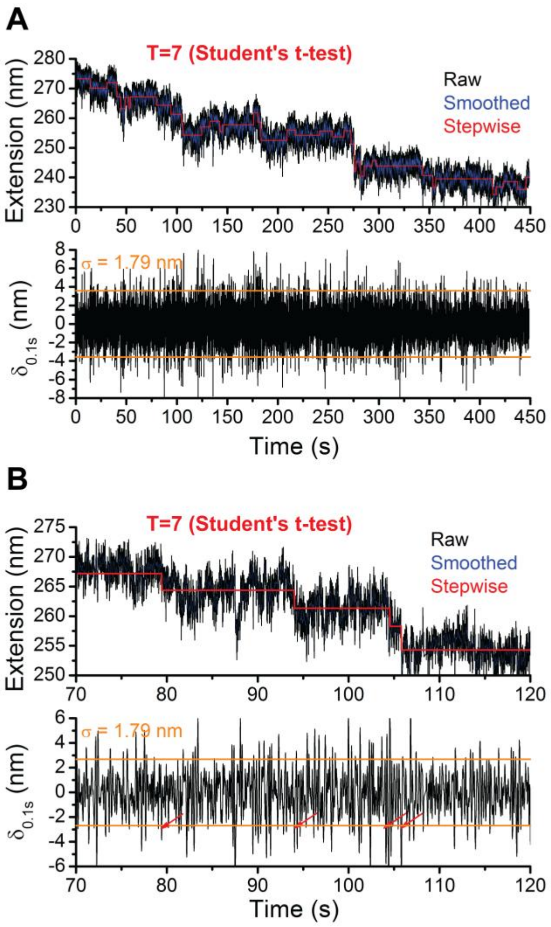

Supplementary Figure S11. Steps detected from the extension time trace of MtRecA filament in the presence of MtRecX. (A). The raw data (black) were smoothed by the

Savitzky-Golay method within 0.2 sec time window (blue). Trial steps are detected when $\Delta_{0.1s}(t) \geq 2\sigma = 3.58$ (orange lines). Only steps with the Student's T-value greater than 7 were accepted as de-polymerization or re-polymerization steps, and the corresponding stepwise time trace was plotted in red. (B) show the zoom-in of (A) from 70-120 second.

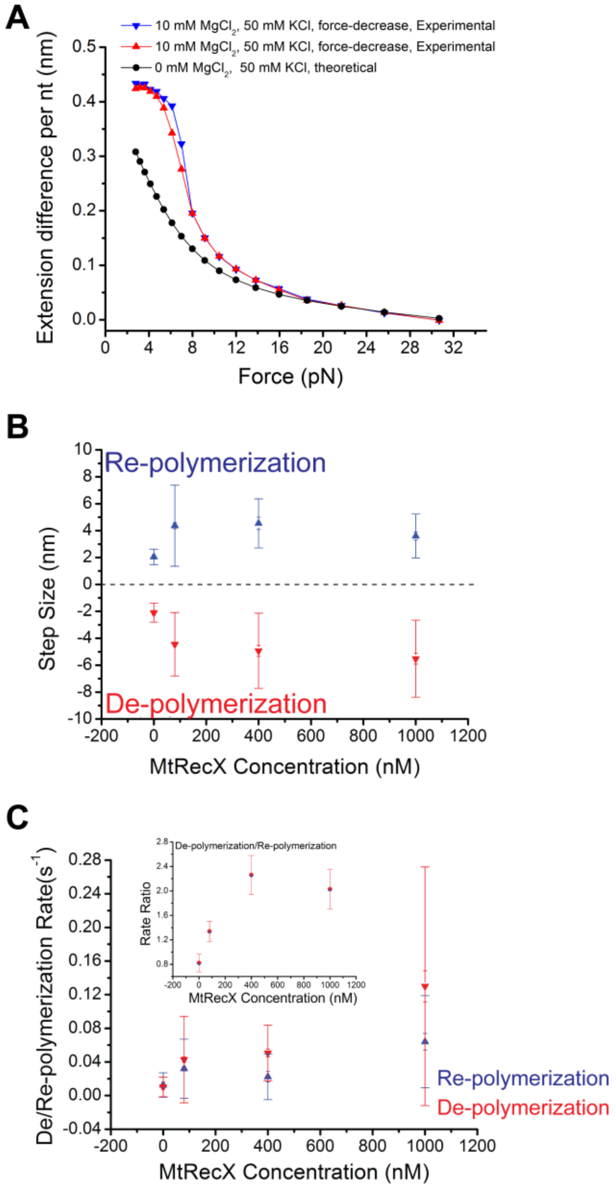

Supplementary Figure S12. Step sizes and rates of de-polymerization or re-polymerization of MtRecA filaments in different concentrations of MtRecX. (A) Extension difference of MtRecA filament and ssDNA. The black circles are estimated

by theoretical models, using naked ssDNA force response with 50 mM KCl and MtRecA filament force response based on the WLC model with a bending persistence length of 1 μm and an estimated contour length of 0.51 nm/nt. The blue tri-angles are data measured in experiments in 50 mM KCl and 10 mM $MgCl_2$. The data are averaged from multiple experiments (N=3). In the force range of 2-4 pN, extension change is ~ 0.3 nm/nt based on theoretical estimation and ~ 0.4 nm/nt based on experimental data. The difference is likely due to the effect of magnesium, which is not considered in the theoretical formula of naked ssDNA force-response. (**B**) Step sizes of de-polymerization (red down-triangles) and re-polymerization (blue up-triangles). (**C**) Rates of de-polymerization (red down-triangles) and re-polymerization (blue up-triangles). The error bars with wider and narrower caps indicate standard deviations (s.d.) and standard errors (s.e.) obtained from multiple (>3) independent experiments, respectively. Insets in (**C**) show the ratio of the rates of de-polymerization and re-polymerization, error bars were obtained from the standard deviations and standard errors of the rates of re-polymerization and de-polymerization through error propagation.

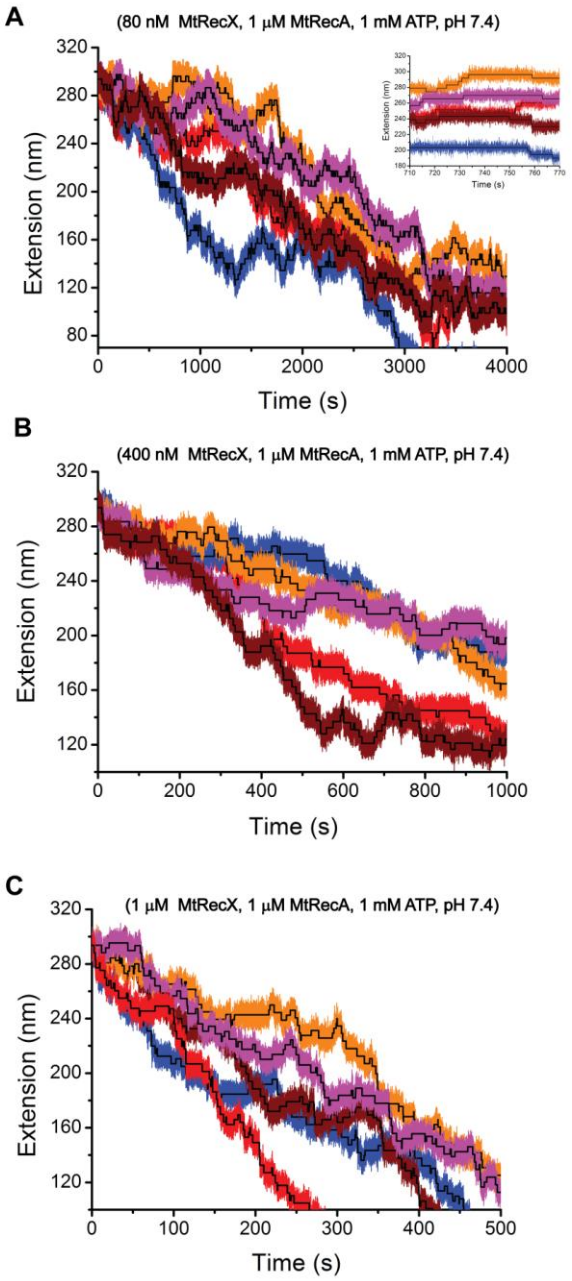

Supplementary Figure S13 Simulated extension evolutions of MtRecA filaments**.** The simulated extension time traces of MtRecA filaments using the kinetics simulation algorithm based on the average values of rates and step sizes of de-polymerization or re-polymerization in Figure S4. Black lines, simulated extension time traces. Gaussian noises with a standard deviation of ~ 4 nm (close to the global standard deviation of the raw time traces in our experiments) are added to each line to mimic the experimental time traces. Five independent simulations at each condition

were plotted.

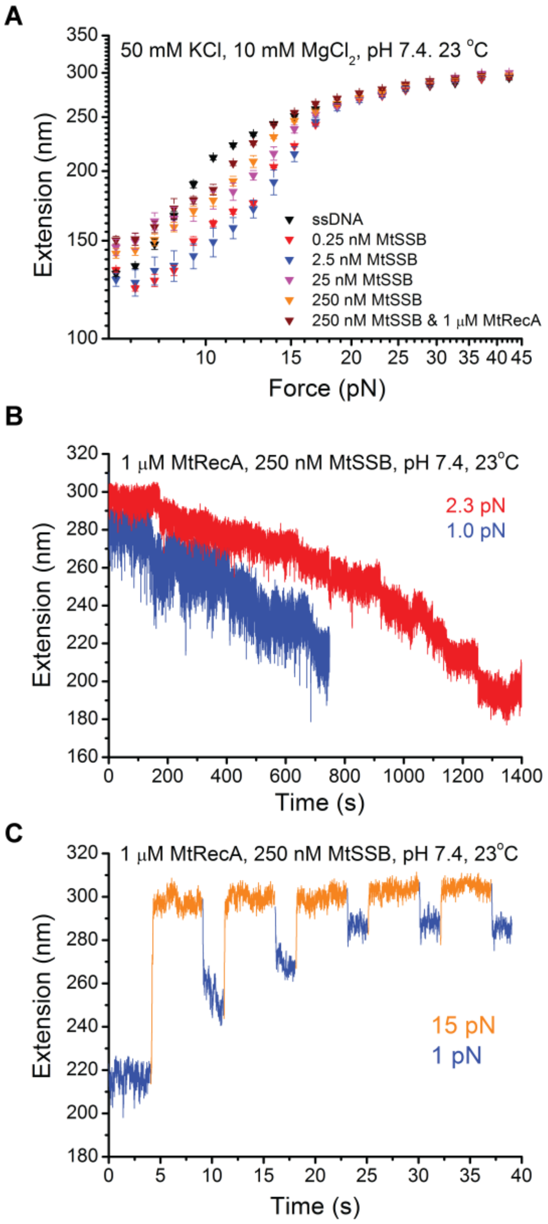

Supplementary Figure S14 Force assisted re-polymerization of MtRecA filament in the presence MtSSB. (A) Force extension curves of ssDNA with different concentrations of MtSSB (0.25 nM – 250 nM), or mixture of MtSSB and MtRecA, indicated by different colors. Symbols and error bars represent the average values and standard deviations of multiple (≥3) repeating force scans at each condition. (B)

De-polymerization of two preformed MtRecA filaments after a mixture of 250 nM MtSSB and 1 μM MtRecA was introduced at low forces < 3 pN. (C) Extension evolution of a partially de-polymerized MtRecA filament during cycles of force-jumping between a low force of ~1 pN and a high force of ~15 pN.

SI-References

1. Chen, H., Fu, H. X., Zhu, X. Y., Cong, P. W., Nakamura, F., and Yan, J. (2011) Improved High-Force Magnetic Tweezers for Stretching and Refolding of Proteins and Short DNA. *Biophys J* **100**, 517-523
2. Fu, H. X., Chen, H., Zhang, X. H., Qu, Y. Y., Marko, J. F., and Yan, J. (2011) Transition dynamics and selection of the distinct S-DNA and strand unpeeling modes of double helix overstretching. *Nucleic acids research* **39**, 3473-3481
3. Zhang, X., Chen, H., Fu, H., Doyle, P. S., and Yan, J. (2012) Two distinct overstretched DNA structures revealed by single-molecule thermodynamics measurements. *Proceedings of the National Academy of Sciences of the United States of America* **109**, 8103-8108
4. Zhang, X., Chen, H., Le, S., Rouzina, I., Doyle, P. S., and Yan, J. (2013) Revealing the competition between peeled ssDNA, melting bubbles, and S-DNA during DNA overstretching by single-molecule calorimetry. *Proceedings of the National Academy of Sciences of the United States of America* **110**, 3865-3870
5. Cui, Y. B., Petrushenko, Z. M., and Rybenkov, V. V. (2008) MukB acts as a macromolecular clamp in DNA condensation. *Nat Struct Mol Biol* **15**, 411-418
6. Cox, J. M., Tsodikov, O. V., and Cox, M. M. (2005) Organized unidirectional waves of ATP hydrolysis within a RecA filament. *PLoS Biol* **3**, e52
7. Bell, J. C., Plank, J. L., Dombrowski, C. C., and Kowalczykowski, S. C. (2012) Direct imaging of RecA nucleation and growth on single molecules of SSB-coated ssDNA. *Nature* **491**, 274-U144
8. Fu, H., Le, S., Chen, H., Muniyappa, K., and Yan, J. (2013) Force and ATP hydrolysis dependent regulation of RecA nucleoprotein filament by single-stranded DNA binding protein. *Nucleic acids research* **41**, 924-932
9. Joo, C., McKinney, S. A., Nakamura, M., Rasnik, I., Myong, S., and Ha, T. (2006) Real-time observation of RecA filament dynamics with single monomer resolution. *Cell* **126**, 515-527
10. Mani, A., Braslavsky, I., Arbel-Goren, R., and Stavans, J. (2010) Caught in the act: the lifetime of synaptic intermediates during the search for homology on DNA. *Nucleic acids research* **38**, 2036-2043
11. Cocco, S., Yan, J., Leger, J. F., Chatenay, D., and Marko, J. F. (2004) Overstretching and force-driven strand separation of double-helix DNA. *Phys Rev E Stat Nonlin Soft Matter Phys* **70**, 011910
12. Smith, S. B., Cui, Y., and Bustamante, C. (1996) Overstretching B-DNA: the elastic response of individual double-stranded and single-stranded DNA molecules. *Science* **271**, 795-799
